# Detection of bacterial DNA in synovial fluid in dogs with arthritis: a comparison between bacterial culture and 16S rRNA polymerase chain reaction

**DOI:** 10.1186/s13028-021-00599-7

**Published:** 2021-08-30

**Authors:** Alexandra Vilén, Bo Nilson, Ann-Cathrine Petersson, Mariana Cigut, Christel Nielsen, Henriette Ström

**Affiliations:** 1 AniCura Landskrona Small Animal Clinic, Föreningsgatan 165, 261 51 Landskrona, Sweden; 2 Department of Clinical Microbiology, Region Skåne, Sölvegatan 23, 223 62 Lund, Sweden; 3 Section of Medical Microbiology, Department of Laboratory Medicine Lund, Lund University, Sölvegatan 23, 223 62 Lund, Sweden; 4 Evidensia Small Animal Referral Hospital of Helsingborg, Bergavägen 3, 254 66 Helsingborg, Sweden; 5 Division of Occupational and Environmental Medicine, Department of Laboratory Medicine, Lund University, 221 00 Lund, Sweden; 6 Öresunds Small Animal Clinic Vellinge, Kompanigatan 27, 235 23 Vellinge, Sweden

**Keywords:** Canine, Incubation, Joint fluid, Paediatric blood culture bottles, Polymerase chain reaction, Septic arthritis, Synovia

## Abstract

**Background:**

Septic arthritis (SA) is a serious condition in dogs that requires a prompt diagnosis and treatment to minimize long-term joint pathology. Although bacterial detection in synovial fluid (SF) through culture or cytology is often performed to confirm diagnosis, the sensitivity of these tests is low. The need for a reliable diagnostic tool to confirm the presence of bacteria in SF in humans has led to the increased use of 16S rRNA (i.e., ribosomal RNA) gene sequencing by polymerase chain reaction (16S rRNA PCR). The aim of this prospective clinical study was to compare the sensitivity and specificity of 16S rRNA PCR with bacterial culture on blood agar plates after pre-incubation of SF in paediatric blood bacterial culture bottles to identify bacteria in dogs with clinical signs of SA and to investigate the usefulness of these methods as diagnostic tools.

**Results:**

Ten dogs with clinical signs of SA, nine with osteoarthritis (OA, control group) and nine with clinical signs of immune-mediated polyarthritis (IMPA, second control group) were examined. Bacterial culture was positive in seven of 10 dogs with clinical SA, of which only two were positive by 16S rRNA PCR. The sensitivity of 16S rRNA PCR and bacterial culture analysis for dogs with clinical SA were 20% and 70%, respectively. All SF samples collected from control group (n = 9) and second control group (n = 14) animals were negative on culture, and 16S rRNA PCR rendered a specificity of 100%**.**

**Conclusions:**

Our study showed a lower sensitivity of 16S rRNA PCR than bacterial culture for dogs with clinical SA. Our findings suggest that there is currently no advantage in using 16S rRNA PCR as a diagnostic tool for dogs with clinical SA. Furthermore, our study indicates that pre-incubation in paediatric blood bacterial culture bottles before bacterial cultivation on blood agar plates might enhance bacterial culture sensitivity compared to other culture methods.

## Background

Septic bacterial arthritis (SA) in dogs is a serious condition that requires early diagnosis and treatment for optimal outcomes. Indeed, delayed or inadequate intervention can lead to irreversible joint destruction and subsequent impaired joint function [1–3].

*Staphylococcus pseudintermedius* is the most commonly isolated causal agent of SA in dogs [1]; other bacteria isolated from SA include beta-haemolytic streptococci, *Staphylococcus aureus*, *Pseudomonas aeruginosa* and *Escherichia coli* [1, 4, 5]. Anaerobic infections have also been reported [6]. Diagnosis of SA is usually based on a combination of clinical and laboratory findings and response to antibiotic therapy [4, 7, 8]. Clinical signs of SA typically include monoarticular pain and lameness, swelling, increased heat over the affected joint, lethargy and pyrexia [1, 4, 8]. Although SA generally presents as monoarthritis, septic polyarthritis can occur, and the clinical presentation might be similar to non-infectious arthritis, such as immune-mediated polyarthritis (IMPA) [1, 9]. Dogs with IMPA can exhibit variable clinical signs, but lameness, joint swelling, and pyrexia are commonly reported. Two or more joints should be affected [10–12]. Despite the usually rapid onset of the clinical signs, a more insidious presentation, such as IMPA, can occur with chronic or low-grade SA [1, 10–13]. Arthrocentesis is essential to differentiate between SA and IMPA, whereby the total white blood cell count (WBC) of the synovial fluid (SF) is usually dramatically increased in dogs with SA, with a predominance of neutrophils [1, 8, 9, 14], as in most cases of IMPA [10, 12, 13]. However, a reliable cut-off for the WBC to distinguish SA from IMPA in dogs or humans has not yet been established [15]. Analysis of SF lactate concentrations has been reported as a useful predictor of SA in both dogs [16] and humans [17]. Nevertheless, the current gold standard for diagnosing SA is the detection of bacteria in samples by cytology or a positive bacterial culture [4, 8]. Unfortunately, the diagnostic yield of these methods is suboptimal [8, 18], as bacteria are reportedly only detected via cytology in 16–54% of dogs [4, 8, 14, 18] and in 19–27% of humans [19] with SA. Moreover, bacterial culture of SF is limited by the time required from sampling until laboratory results are ready, which is usually in the range of 3–5 days, and false negative results occur in both dogs [1, 3, 4, 8, 18] and humans [20–24], at approximately 50% in the former (range of 20–50%). Several reasons for this have been implicated, such as the recent use of antimicrobials, fastidious nature of the causative bacteria and method of sample collection [1, 3, 22]. Pre-incubation of synovia in blood culture bottles has been suggested to increase the detection sensitivity of bacteria in SF [3, 24]. To our knowledge, there are no studies on the use of paediatric blood culture bottles for diagnosing dogs. Compared to regular bottles, these bottles are adjusted for smaller sample volumes, which may be advantageous because the sample volume for dogs may be small [4, 8]. Additionally, there are several reports of bacterial detection and identification by 16S rRNA polymerase chain reaction (PCR) for the diagnosis of SA in humans [21, 22, 25–28], with the advantage that PCR analysis does not require live bacterial culture but only bacterial DNA [20–22, 25, 26, 29–32].

Overall, there are few studies of PCR as a diagnostic tool for SA in veterinary medicine. In a study by Elmas et al. on bacterial detection in horses with septic synovitis, the sensitivity of 16S rRNA PCR was significantly higher than that of bacterial culture [33]. Other studies have not found a significant difference between 16S rRNA PCR and bacterial culture for horses [34] or dogs [18] with SA.

The aim of this prospective clinical study was to compare the sensitivity and specificity of 16S rRNA PCR with bacterial culture on blood agar plates after pre-incubation of synovia in paediatric blood bacterial culture bottles to identify bacteria in dogs with clinical signs of SA and to investigate the usefulness of these methods as diagnostic tools. We hypothesised that 16S rRNA PCR would be more sensitive than the bacterial culture method used to detect bacteria in SF from dogs with SA. We further hypothesised that 16S rRNA PCR would have a higher specificity than bacterial culture.

## Methods

### Criteria for selection of cases

Dogs presenting at Evidensia Small Animal Referral Hospital Helsingborg, Sweden, from November 2010 to November 2013 with clinical signs of SA or IMPA were considered for this study. Dogs that were admitted for joint surgery due to degenerative osteoarthritis (OA) were considered controls, as they usually have low to normal cell counts in synovia [35]. IMPA cases were included as a second control group because of the similar clinical features to SA cases [10–13]. The inclusion criteria for the SA group were clinical signs of infectious monoarthritis (monoarticular pain and lameness, swelling with or without lethargy and/or pyrexia [1, 4, 8]) in combination with elevated leucocytes (≥ 20 × 10^9^ cells/mL) and neutrophils (≥ 20%) in SF [1, 4, 35]. Inclusion criteria for the control group were signs of mono- or polyarthropathy due to degenerative disease and low SF leucocyte (< 5 × 10^9^ cells/mL) and neutrophil (< 10%) counts [35]. For the secondary control group (IMPA), inclusion criteria were clinical signs of inflammatory polyarthritis (lameness and joint swelling of two or more joints with or without pyrexia [10–13]) in combination with elevated SF leucocytes (≥ 5 × 10^9^ cells/mL) and neutrophils (≥ 12%) in ≥ 2 joints [10–13]. A further requirement for inclusion in all groups was that at least 0.8 mL synovia could be collected from a minimum of one joint of each patient to obtain enough SF for cytology, bacterial culture and 16S rRNA PCR. Exclusion criteria were other serious illnesses, such as signs of septic polyarthritis.

Data regarding breed, weight, sex, age, duration of symptoms, affected joints, prior surgery within 6 months, and prior treatment with antimicrobials within 6 months were collected. A total of 10 dogs were found to meet the inclusion criteria for the SA group, with nine each for the control group and second control group.

### Collection of SF

Arthrocentesis was performed while the dogs were under general anaesthesia and using strict aseptic conditions. A minimum of 0.2 mL of the collected SF intended for 16S rRNA PCR was immediately transferred to a sterile container, which was placed at − 20 °C within 30 min. Additionally, at least 0.2 mL SF was transferred to an EDTA tube (S-Monovette® K3 1.2 mL, Sarstedt, Germany) for cytological examination (Gram stain) and a manual cell count (Bürker chamber, 25 μL SF diluted in 475 μL saline). A minimum of 0.3 mL SF was injected into a paediatric blood bacterial culture bottle containing medium enriched with soybean-casein digest broth and CO_2_ (Bactec Peds Plus™, Becton Dickinson and Company, Sparks, MD, USA). The syringe was flushed with culture medium before the needle was withdrawn from the bottle. The blood culture medium was incubated at 37 °C for 19–24 h before inoculation onto a blood agar plate (500 mL blood agar base (CM0055B, Oxoid™, Thermo Fisher Scientific Inc., UK) with 16 mL bovine blood in sodium citrate, Håtunalab AB, Bro, Sweden) [3]. The blood agar plate was subsequently incubated at 37 °C for 24 h; negative cultures were incubated for another 24 h. The collected and frozen SF samples for DNA analyses were stored at − 20 °C until transport (in − 20 °C) to a clinical microbiologic laboratory (Clinical Microbiology, Region Skåne, Lund). The samples were stored for a maximum of 6 months before they were sent in bulk. Biomedical analysts employed at Evidensia Small Animal Referral Hospital Helsingborg performed all cytological examinations of joint fluid as well as the bacterial cultures and susceptibility testing.

### Broad-range PCR

SF was subjected to 16S rRNA gene PCR. The analysis was performed as previously described [36]. DNA was extracted from 200 µL fluid using a Bio Robot EZ-1 with DNA Tissue Kit (Qiagen, Hilden, Germany) after treatment with Proteinase K according to instructions by the manufacturer. Amplification was carried out in 50 µL of reaction mixture containing 1 × PCR buffer (Qiagen), 2 mM MgCl_2_, 200 µM of each dNTP, 1.25 U HotStar Taq DNA polymerase (Qiagen), 10 pmol of each primer and 5 µL template. P515f (5′-TGC CAG CMG CCG CGG TWA T-3′) [37] and P1067r (5′-AAC ATY TCA CRA CAC GAG CT-3′) [36] were used as primers for PCR and sequencing. A pre-PCR step of 15 min at 95 °C was followed by 40 cycles of 93 °C for 50 s, 52 °C for 50 s and 72 °C for 50 s and a final step of 5 min at 72 °C. Tubes with no target DNA and *Haemophilus influenzae* DNA were included as negative and positive controls, respectively. Both strands of the approximately 520-bp PCR product were sequenced using BigDye Terminator Cycle Sequencing Kit (Applied Biosystems Inc., Foster City, USA) and analysed using an ABI PRISM 3100 Genetic Analyzer (Applied Biosystems Inc. by BM-Labbet, Furulund, Sweden). For species identification, sequences were compared to databases available at the National Centre for Biotechnology Information using the BLAST similarity search program (www.ncbi.nlm.nih.gov).

### Blood sample

A complete blood cell count (CBC) was performed for all dogs. C-reactive protein (CRP) was analysed in 8/10 dogs in the SA group and 5/9 dogs in the second control group; CRP was not analysed in the control group.

### Statistical methods

Data were not normally distributed, and descriptive statistics are thus presented as the median and interquartile range. Statistical comparison of groups was performed using non-parametric Kruskal–Wallis and Mann–Whitney U tests.

The sensitivity and specificity of bacteriological culture and 16S rRNA PCR were calculated according to inclusion criteria in each set of dogs. Clinical symptoms and synovial cell counts were used to establish the gold standard for each group. Associated confidence intervals were constructed using a binomial distribution to reach exact estimates. Variables indicating whether bacteriological culture and 16S rRNA PCR correctly classified each joint as positive or negative according to the gold standard were created and compared using the McNemar test for paired samples. The mid-p version of the McNemar test [38] was chosen, as the number of discordant pairs was low.

Statistical significance was set at P < 0.05. All analyses were performed using SAS Enterprise Guide 6.1 (SAS Institute Inc., Cary, NC, USA).

## Results

### SA

Most of the dogs were large to giant breeds based on weight (Table 1). The median duration of clinical signs was 2.5 days (ranging from 0 to 365 days). The most affected joints were the elbow (n = 3) and stifle (n = 3), followed by the shoulder (n = 2), hock (n = 1) and carpus (n = 1). Four dogs had undergone surgery on the affected joint less than 6 months prior. One joint was infected by a bite wound. Four dogs had a history of treatment with antibiotics in the last 6 months.

**Table 1:** Clinical characteristics

	Septic arthritis n = 10	Second control group (IMPA) n = 9	Control group (OA) n = 9
Age (months)	38 (26–90)^a^	61 (51–72)	12 (11–42)
Weight (kg)	42 (30–47)	33 (30–35)	39 (26–45)
Blood sample			
White blood count (× 10^9^ cells/L)	12 (6–18)	16 (11–19)	9 (6–10)
CRP (mg/L)	n = 8 72 (39–110)	n = 5 89 (55–97)	Not evaluated
Synovial fluid	n = 10	n = 27	n = 9
Leucocytes (× 10^9^ cells/L)	125 (65–150)	58 (34–90)	0 (0–1)
Neutrophils (%)	88 (72–93)	86 (74–89)	1 (0–3)
Small monocytes (%)	1 (0–3)	2 (1–7)	2 (1–7)
Large monocytes (%)	12 (5–19)	11 (8–19)	96 (81–97)

n: Number; CRP: Canine reactive protein

^a^Median (inter-quartile range)

Clinical characteristics (signalment, blood parameters and synovial fluid cell count) of the three different groups included, i.e., dogs with clinical signs of septic arthritis, dogs with clinical signs of immune-mediated polyarthritis (IMPA, second control group) and the control group of dogs with signs of osteoarthritis (OA)

Positive bacterial cultures were obtained in seven cases, with four different bacterial isolates identified. Positive 16S rRNA PCR was obtained in two cases, both of which were also positive by bacterial culture (*Streptococcus* spp.) (Table 2). Positive bacterial cultures but negative 16S rRNA PCR results were obtained for the four dogs treated with antimicrobials.

**Table 2:** Microbiological data

Pathogen	Positive by bacterial culture	Positive by PCR
Beta-haemolytic streptococci	3	2
*Staphylococcus pseudintermedius*	2	0
*Staphylococcus aureus*	1	0
*Pasteurella* spp.	1	0

PCR: 16S rRNA polymerase chain reaction

Number of positive bacterial cultures and 16S rRNA polymerase chain reaction analyses for dogs with signs of septic arthritis. Seven of 10 dogs with signs of septic arthritis had a positive bacterial culture, two of which were also positive by 16S rRNA polymerase chain reaction analysis

### Control group

Nine dogs were included in the control group. Most of the dogs were large to giant breeds based on weight (Table 1). SF was collected from the elbow (n = 7), stifle (n = 1) and shoulder (n = 1). Bacterial culture and 16S rRNA PCR were performed for all nine cases, and the results were negative.

### Second control group

Nine dogs were included in the second control group (IMPA), and most were large to giant breeds based on weight (Table 1). The median duration of clinical signs was 1 day (range 0–10 days). A total of 27 joint taps were performed (hock n = 14, carpus n = 8, stifle n = 3, elbow n = 2). None of the dogs had been treated with systemic antimicrobials in the last 6 months.

A manual cell count was performed on 26/27 samples (for one case, there was only enough material for a smear). Bacterial culture and PCR were performed for 14/27 samples, and both were negative for all nine dogs. Although a few solitary gram-positive cocci were identified in one culture, there were too few to be classified to the species level culture, and the laboratory report noted that their presence was of no clinical relevance.

CRP was elevated in all dogs tested (n = 8) with clinical signs of SA and in 4 of 5 dogs with clinical signs of IMPA (Table 1). There was no significant difference in CRP between the dogs diagnosed with SA and those diagnosed with IMPA (P = 0.94 Mann–Whitney test).

This study found 16S rRNA PCR to be a less sensitive diagnostic method for detecting bacteria in SF in dogs with SA compared to the bacterial culture method. The sensitivity of 16S rRNA PCR to detect bacteria in SA was only 20% (2/10 dogs) compared to 70% (7/10 dogs) for bacterial culture. Furthermore, the combination of 16S rRNA PCR and culture did not improve sensitivity. The specificity of 16S rRNA PCR and the culture method were both 100%, as all analysed joints in the two control groups were negative (Table 3).

**Table 3:** Statistical comparison

	Sensitivity (95% CI)	Specificity (95% CI)
Bacterial culture	70 (35–93)	100 (85–100)
PCR	20 (3–56)	100 (85–100)
Bacterial culture + PCR	70 (35–93)	100 (85–100)

CI: Confidence interval are exact based on the binomial distribution

PCR: 16S rRNA polymerase chain reaction

Statistical comparison of sensitivity and specificity of bacterial culture and 16S rRNA polymerase chain reaction and the combination of bacterial culture and 16S rRNA polymerase chain reaction to demonstrate bacteria in the synovial fluid in dogs with suspected septic arthritis

## Discussion

To our knowledge, a very limited number of studies applying16S rRNA analysis for SF from dogs have been performed. The sensitivity of 16S rRNA PCR for diagnosing SA in our study was 20%. This was considerably lower than previously published sensitivities in veterinary medicine (73.7 to 89.5%) [18, 33, 34]. In humans, sensitivity ranges from 23.5 to 96.2%, and there are conflicting reports in the literature regarding whether 16S rRNA PCR improves diagnostics when diagnosing SA [21, 22, 27].

In our study, we employed a well-established primer pair for 16S rRNA [36] used by many clinical laboratories for human diagnostics. To our knowledge, no previous study has evaluated this primer pair for use in SF from dogs.

There are several factors that might explain the low sensitivity we observed. 16S rRNA PCR involves several critical steps, such as DNA extraction and PCR amplification, and false negative results are reportedly not uncommon, especially if only a few bacteria are present [30, 39–42].

To avoid contamination during extraction of DNA, which could cause false positive results, procedures following good laboratory practice for molecular identification of bacterial DNA were applied in this study. Proteinase K was used in the process of DNA extraction, which is common in DNA extraction protocols to avoid contamination resulting in false positive results. Proteinase K digests contaminating proteins and protects nucleic acids from degrading enzymes [30]. Nonetheless, contaminating DNA is present in many reagents used for DNA extraction, and it has been suggested that it is not possible to eliminate DNA contamination without a significant decrease in sensitivity [41].

SF contains PCR inhibitors that may cause false negative results. This is due to the natural compounds in SF as well as the high viscosity in combination with the ionic and macromolecular contents [30]. Unfortunately, the processes used to remove these inhibitors may also destroy or dilute any bacteria present [23, 30].

The Qiagen column we used has a limited capacity to bind DNA; in human patients with sepsis, it has been suggested that leucocyte DNA might compete with bacterial DNA, which could lead to false negative results [43]. All dogs in the SA group had a high leucocyte count in SF (median 125 × 10^9^ cells/mL, range 65–150), which might explain why bacterial DNA was not identified as expected in the present study.

Antimicrobial treatment has been suggested to inhibit bacterial culture as well as PCR analysis, but this does not seem to explain our results. Four dogs in the SA group received antimicrobials within 6 months prior to SF sampling. All of them were positive by culture but negative by 16S rRNA PCR.

Protocols regarding the collection, handling, storage and transport of samples by the human clinical microbiology laboratory that performed all 16S rRNA PCR analyses in our study were followed, and there were no known deviations from these protocols. Nevertheless, failures in methodology need to be considered, as the correct handling of samples after collection is critical when using molecular techniques [41]. All samples for 16S rRNA PCR analysis were frozen within 30 min after collection and stored at − 20 °C. Freezing clinical samples is considered standard and often performed prior to DNA analysis [33, 41], but there are some studies on the effect of storage on bacterial content in SF. For example, Carlsen et al. showed that the *Mycoplasma genitalium* DNA load decreased after storage at − 20 °C for up to 18 months, which was especially notable with clinical specimens compared to frozen DNA extracts [44]. In addition, freezing aqueous solutions of DNA samples at − 20 °C might have a negative effect on DNA stability [45]. In our study, DNA was not extracted before storage, and this cannot be ruled out as having a negative effect on our test material.

There are several limitations in our study. The sample size was small. The difficulty in obtaining a larger number of dogs with SA might be due to a combination of SA being uncommon and because some of the dogs presented with clinical signs of SA, with cytological findings that supported the diagnosis but an insufficient total sample volume. All samples in this study were collected by the veterinary surgeon on duty, with varying experience in performing a joint tap, as SA is a clinical emergency. Low sample volume in dogs with SA has previously been reported in a study where samples could not be obtained in 23.5% of cases, even by an experienced surgeon [4].

All samples in the control group were negative by both culture and 16S rRNA PCR. We included a second control group of dogs with IMPA in addition to the control group to further rule out false positive samples. Earlier studies of 16S rRNA PCR on SF in dogs have described the presence of bacteria based on 16S rRNA PCR analyses in normal stifles of dogs as well as in dogs with stifle pathology traditionally considered to be non-infectious [46–49]. Additionally, a wide variety of bacteria with uncertain significance have been identified in in human patients with rheumatoid arthritis, mostly comprising gut and skin commensals, as well as some species not previously described [50, 51]. All cultured samples in the second control group were negative except for one in which a small number of solitary cocci were identified. This finding was discarded by the laboratory as contamination and considered to have no clinical relevance. All analysed samples in the two control groups were negative for 16S rRNA PCR, yielding a specificity of 100%.

In future studies on 16S rRNA PCR sensitivity in dogs with SA, it would be valuable to compare the different published 16S rRNA primer pairs to see if any pair result in higher sensitivity than the others. It would also be valuable to obtain a larger sample size and a confirmed diagnosis of SA. Unfortunately, as previously mentioned, a cut-off value for the number of WBCs in synovia has not been established to separate infectious from inflammatory, non-infectious arthritis [15]. This correlated well with our findings, as there was no significant difference in WBC between dogs with SA and dogs with IMPA (P = 0.35). The same conclusion was made regarding CRP, with no significant difference between these two groups of dogs (P = 0.94). Analysis of SF lactate concentration in our study might have helped to more clearly define cases of suspected SA [16].

The current gold standard to diagnose SA is the detection of bacteria in samples by cytology or a positive bacterial culture [4, 8]. In our study, bacterial culture of SF was positive in 70% of dogs with presumed SA. This is considerably higher than the usually reported sensitivity of approximately 50% [3, 52]. Pre-incubation of synovia in blood culture bottles before culture on blood agar plates has been suggested to improve results [3, 24], but the results are not consistent [52]. The improved sensitivity in our study is thought to be due to the use of pre-culture incubation of synovia in paediatric blood culture bottles, instead of in standard bottles, before culture on blood agar plates. The use of paediatric blood culture bottles (Bactec Peds Plus™, Becton Dickinson and Company, Sparks, MD, USA) requires smaller sample volumes than with standard bottles. Small sample volumes are, as previously mentioned, common for dogs [4]. By using complete bottle culture systems instead of agar plates, the sensitivity may improve even more. In a recent study by Cohen et al., culture in bottles (Bactec**®** method) showed growth in 113/148 cases (76.4%) compared to 96/154 (62.3%) with agar plate cultures [24].

Although 16S rRNA PCR may allow for a more rapid result regarding the presence of bacteria in synovia [21, 22, 33], more studies are needed before this analysis can be considered routine for the diagnosis of SA in dogs. To be a useful diagnostic tool, 16S rRNA PCR must have a high sensitivity and be reliable. The advantage of a positive bacterial culture is not only the confirmation of bacteria but also the possibility of obtaining a susceptibility report that can ultimately lead to the best treatment plan.

## Conclusion

The main finding in this study is that the sensitivity of 16S rRNA PCR was considerably lower than that of the bacterial culture method used. Currently, there is not enough evidence to routinely include 16S rRNA PCR in the diagnostic work-up for dogs with suspected SA. We further conclude that pre-incubation in paediatric blood bacterial culture bottles prior to agar plate culture improves sensitivity and should be considered a routine method for these patients.

## Data Availability

The data sets during and/or analysed during the current study are available from the corresponding author upon reasonable request.
